# Nuclear receptor coactivator 6 promotes HTR‐8/SVneo cell invasion and migration by activating NF‐κB‐mediated *MMP9* transcription

**DOI:** 10.1111/cpr.12876

**Published:** 2020-08-13

**Authors:** Liang Wu, Kun‐qing Zhao, Wei Wang, Li‐na Cui, Lin‐li Hu, Xiang‐xiang Jiang, Jun Shuai, Ying‐pu Sun

**Affiliations:** ^1^ Reproductive Medical Center The First Affiliated Hospital of Zhengzhou University Zhengzhou Henan China; ^2^ Henan Key Laboratory of Reproduction and Genetics The First Affiliated Hospital of Zhengzhou University Zhengzhou Henan China; ^3^ Henan Provincial Obstetrical and Gynecological Diseases (Reproductive Medicine) Clinical Research Center The First Affiliated Hospital of Zhengzhou University Zhengzhou Henan China; ^4^ State Key Laboratory of Stem Cell and Reproductive Biology Institute of Zoology Chinese Academy of Sciences Beijing China; ^5^ Department of Reproductive Medicine The Second Hospital of Hebei Medical University Shijiazhuang Hebei China

**Keywords:** invasion and migration, MMP9, NCOA6, NF‐κB, placental trophoblast

## Abstract

**Objectives:**

NCOA6 is a transcription coactivator; its deletion in mice results in growth retardation and lethality between 8.5 and 12.5 dpc with defects in the placenta. However, the transcription factor(s) and the mechanism(s) involved in the function of NCOA6 in placentation have not been elucidated. Here, the roles of NCOA6 in human cytotrophoblast invasion and migration were studied.

**Materials and Methods:**

Human placenta tissues were collected from normal pregnancies and pregnancies complicated by early‐onset severe preeclampsia (sPE). Immunofluorescence, RT‐qPCR and Western blotting were used to determine *NCOA6* expression. Transwell invasion/migration assays were performed to explore whether *NCOA6* knockdown affected human placenta‐derived HTR‐8/SVneo cell invasion/migration. Gelatin zymography was performed to examine the change in the gelatinolytic activities of secreted MMP2 and MMP9. Luciferase reporter assays were used to explore whether NCOA6 coactivated NF‐κB‐mediated *MMP9* transcription.

**Results:**

NCOA6 is mainly expressed in the human placental trophoblast column, as well as in the EVTs. HTR‐8/SVneo cell invasion and migration were significantly attenuated after *NCOA6* knockdown, and the secretion of MMP9 was decreased due to transcriptional suppression. NCOA6 was further found to coactivate NF‐κB‐mediated *MMP9* transcription. Moreover, expression of *NCOA6* was impaired in placentas of patients complicated by early‐onset sPE.

**Conclusions:**

Thus, we demonstrated that NCOA6 is important for cytotrophoblast invasion/migration, at least partially, by activating NF‐κB‐mediated *MMP9* transcription; the downregulation of *NCOA6* may contribute to the pathogenesis of early‐onset sPE.

## 1 INTRODUCTION

Normal placentation is indispensable for foetal development. Two major types of differentiation exist in trophoblasts during human placentation, which include trophoblast invasion and syncytialization. During invasion, cytotrophoblasts (CTBs) proliferate to form the trophoblast column (TC), from which they differentiate into the invasive extravillous trophoblasts (EVTs) that either invade into the maternal decidua/myometrium to anchor the placenta (as interstitial EVTs) or invade and remodel maternal spiral arteries to improve maternal blood flow as required for pregnancy (as endovascular EVTs). During syncytialization, mononucleated CTBs fuse to become multinucleated syncytiotrophoblasts (STBs).^1^ Abnormal trophoblast differentiation and subsequent functional impairment result in pregnancy‐related diseases, including preeclampsia (PE) due to shallow interstitial EVT invasion into the maternal decidua/myometrium and incomplete remodelling of maternal vessels caused by impaired endovascular EVT invasion into blood vessels.^2^, ^3^, ^4^

*N*uclear receptor *coa*ctivator *6* (*NCOA6*) was first identified in human breast tumours and was originally named *A*mplified *i*n *b*reast *3* (*AIB3*).^5^, ^6^ In addition to gene amplification in breast cancer, *NCOA6* was also found to be amplified and overexpressed in colon cancers and lung cancers.^7^ As a transcription coactivator, NCOA6 plays multiple roles by coactivating specific transcription factors, including PPARα,^8^ PPARγ,^9^ AP‐1, CRE and NF‐κB.^10^ Mice with *Ncoa6* deletion showed growth retardation and lethality between 8.5‐12.5 days post‐conception (dpc), most likely due to developmental defects in the placenta.^9^, ^11^, ^12^ Since both the labyrinth and spongiotrophoblast layers of the placenta in *Ncoa6* ^−/−^ mice were severely affected, the essential roles of NCOA6 might be the coactivation of key transcription factors in placentation.^9^, ^11^ However, the transcription factor(s) and the mechanism(s) involved in the function of NCOA6 in placentation have not been elucidated.

In this study, we explored the functions of NCOA6 in human trophoblast invasion and migration. NCOA6 was mainly expressed in the TC and EVT of human placentas. *NCOA6* knockdown significantly suppressed the invasion and migration of human placenta‐derived HTR‐8/SVneo cells with reduced matrix metalloproteinase 9 (MMP9) secretion. Luciferase reporter assays were further used to test whether NCOA6 contributed to NF‐κB‐mediated *MMP9* transcription. In addition, the transcription of *NCOA6* in placentas from patients with early‐onset sPE, showing inadequate trophoblast invasion and subsequent incomplete remodelling of maternal spiral arteries, was found to be impaired. Thus, we demonstrated that NCOA6 promotes the invasion and migration of HTR‐8/SVneo cells, at least partially, by coactivating NF‐κB‐mediated *MMP9* transcription.

## 2 MATERIALS AND METHODS

### 2.1 Human placenta collection

Placental villi from the first trimester (6‐8 weeks of gestation, n = 3) were sampled from normal pregnancies after legal abortion; placental tissues from the second trimester (17‐21 weeks of gestation, n = 3) were collected after inevitable abortions that had been accidentally caused by external hurt; the third‐trimester placenta samples were obtained from normal pregnancies (normal group, between 36 and 40 weeks of gestation; n = 15) and pregnancies complicated by early‐onset sPE (early‐onset sPE group, between 33 and 37 weeks of gestation; n = 13). All tissues were sampled with informed consent at the Department of Gynecology and Obstetrics in the First Affiliated Hospital of Zhengzhou University. Individuals with early‐onset sPE were recruited as previously reported, without any other maternal complications.^13^, ^14^ The clinical characteristics of all the pregnant women enrolled in this study are listed (Table 1), and the protocol for sample collection was authorized by the Ethics Committee of the First Affiliated Hospital of Zhengzhou University (2019‐KY‐288). Six small tissue blocks (~0.2 cm^3^ each) were collected randomly from the foetal side of each third‐trimester/term placenta to achieve uniform sampling and avoid contamination of maternal tissue, followed immediately by snap‐freezing and storage in liquid nitrogen within 30 minutes of caesarean birth.

**Table 1:** Clinical characteristics of the pregnant women enrolled in this study

Characteristics	Normal (n = 15)	Early‐onset sPE (n = 13)	*P* value
Maternal age (y)	29.8 ± 2.7	29.8 ± 4.0	.962
Pre‐pregnancy body mass index (kg/m^2^)	21.5 ± 2.0	22.9 ± 2.0	.123
Systolic blood pressure (mm Hg)	114.9 ± 9.9	159.0 ± 18.0*	<.001
Diastolic blood pressure (mm Hg)	73.8 ± 7.9	99.9 ± 11.8*	<.001
Proteinuria (g/24 h)	Normal/non‐detected	4.5 ± 2.3*	<.001
Primiparae (n)	10 (66.7%)	8 (61.5%)	NA
Current smoker (n)	0 (0%)	0 (0%)	NA
Han ethnicity (n)	15 (100%)	13 (100%)	NA
Female foetus (n)	8 (53.3%)	9 (69.2%)	NA
Gestational age at delivery (wk)	38.4 ± 1.2	34.5 ± 1.5*	<.001
Birth weight (g)	3241.6 ± 467.8	2076.2 ± 385.4*	<.001

Note

Values are expressed as the mean ± SD, and statistical analyses were performed by using independent‐samples *t* test in SPSS Statistics 17.0.

Abbreviations: NA, not analysed; sPE, severe preeclampsia.

* Compared to normal pregnancy, *P* < .001.

### 2.2 Paraffin‐embedded section preparation and immunofluorescence

Paraffin‐embedded sections of placental tissue were prepared as previously reported.^15^ After antigen retrieval and blocking, the serial sections were treated with the following primary antibodies overnight at 4°C: rabbit anti‐human NCOA6 serum that was generously provided by Professor Jian‐ming Xu at Baylor College of Medicine in USA and Professor Hong‐mei Wang at Institute of Zoology, CAS in China (1:500),^16^ mouse anti‐human Cytokeratin 7 (CK 7, 180234, Invitrogen; 1:200), mouse anti‐human HLA‐G (sc‐21799, Santa Cruz; 1:200) and mouse anti‐human vimentin (MA5‐11883, Invitrogen; 1:200). After incubations with secondary antibodies: Alexa Fluor Plus 647‐conjugated antibody (A32733, Invitrogen) for NCOA6 and Alexa Fluor 488‐conjugated antibody (A‐11001, Invitrogen) for others, the serial sections were finally treated with DAPI (R37606, Invitrogen) in Vectashield^®^ antifade mounting medium (Vector Laboratories). Florescence images were obtained with a LSM 780 confocal laser‐scanning microscope (ZEISS). All photographs were arranged and processed by PowerPoint (Microsoft Office Professional Plus 2010, Microsoft Corporation) and Photoshop CS6 (Adobe Systems).

### 2.3 Primary CTB isolation and subsequent in vitro induction of EVT

Primary CTBs were isolated as previously reported.^17^ For the in vitro differentiation of primary CTBs into EVTs, 2.5 × 10^6^ CTBs were placed into 35‐mm dishes precoated with 1 mL of liquid Matrigel (BD Biosciences) at 1 mg/mL and maintained in DMEM/F12 (Gibco) containing 10% foetal bovine serum (FBS, Gibco) for 48 hours.

### 2.4 Cell culture

The trophoblast cell line HTR‐8/SVneo, a generous gift from Professor C. H. Graham (Queen's University, Canada), was cultured as previously reported.^14^, ^18^ Plasmocin^™^ prophylactic (5 μg/mL; InvivoGen) was also added to the culture medium to avoid mycoplasma contamination. Mycoplasma contamination detection was performed every month by a Mycoplasma Detection Kit (Yeasen). The HTR‐8/SVneo cell line has never been listed in the Database of Cross‐Contaminated or Misidentified Cell Lines maintained by ICLAC and NCBI Biosample.^19^

### 2.5 Plasmid construction

The pGL3‐Basic‐*MMP9* promoter luciferase reporter (inserted with a 726‐bp *MMP9* proximal promoter fragment) and its mutant vector M1 were both generous gifts from Professor Anthony J. Valente (University of California, USA).^20^ The *MMP9* promoter mutant vectors M2 and M3 were constructed with a Gibson Assembly Cloning Kit (NEB) and confirmed by complete nucleotide sequencing (Invitrogen). The pRenilla‐TK vector was a generous gift from Professor Qiang Wang (Institute of Zoology).^21^ The vector for the ectopic expression of NCOA6 was kindly provided by Professor Jian‐ming Xu (Baylor College of Medicine) and Professor Hong‐mei Wang (Institute of Zoology). The vector for the ectopic expression of RELA/p65 was kindly provided by Professor Qin‐miao Sun (Institute of Zoology).

### 2.6 siRNA and plasmid transfections

Stealth RNAi^™^ siRNAs that interfere with *NCOA6* (HSS118106, HSS118107 and HSS177130) and an unconjugated Med GC negative control (12935300) were both bought from Invitrogen. Plasmid and siRNA transfections in HTR‐8/SVneo cells were performed as previously reported.^14^ Among the set of three Stealth RNAi™ siRNAs targeting *NCOA6*, two (HSS118106 and HSS177130) that significantly interfered *NCOA6* expression and decreased HTR‐8/SVneo cell invasion and migration (data not shown) were pooled together to transfect HTR‐8/SVneo cells to exclude potential off‐target effects.

### 2.7 Transwell invasion/migration assays

Transwell invasion/migration assays were carried out as previously reported.^14^ 1.0 × 10^5^ HTR‐8/SVneo cells were added without FBS to the upper compartment of Corning Costar^®^ transwell inserts pretreated with Matrigel (60 μL of 1 mg/mL for invasion assays) or not (for migration assays). After 22 hours of culture, all of the invaded/migrated cells outside the insert membrane were stained with haematoxylin and photographed. The number of invaded/migrated cells after the knockdown of *NCOA6* was normalized to that from the negative control treatment.

### 2.8 Cell counting kit‐8 (CCK‐8) assay

CCK‐8 incubations were carried out with CCK‐8 reagent (Dojindo Laboratories) 0, 24, 48 and 72 hours after cell‐seeding as previously reported.^14^ The cell medium was renewed every day to ensure sufficient growth nutrition for plated cells. The absorbance at 450 nm of CCK‐8 incubation medium was measured by a Varioskan^™^ Flash Microplate Reader (Thermo Scientific).

### 2.9 Gelatin zymography

Gelatin zymography was carried out by using an MMP zymography assay kit (Applygen). Before the cells that invaded through the insert membrane in the invasion assays were fixed, 20 μL cell medium inside the insert was mixed with 2× SDS‐PAGE non‐reducing buffer, loaded on a gel (20 μL each) and separated via 10% SDS‐PAGE containing 1.0 mg/mL gelatin. After Buffer A rinsing, Buffer B incubation and R‐250 staining, bands representing the gelatinolytic activities of secreted MMP2 and MMP9 were recorded with a ChemiDoc MP System (Bio‐Rad).

### 2.10 RT‐qPCR

RT‐qPCR assays were carried out as previously reported.^14^ Total RNA extracted by TRIzol reagent (Life Technologies) was applied in the RT reaction (Takara). Quantitative PCR was performed by using a QuantiNova^®^ SYBR Green PCR Kit (QIAGEN) in a QuantStudio 5 Real‐Time PCR System (Applied Biosystems). *NCOA6* expression was examined by using the corresponding glyceraldehyde‐3‐phosphate dehydrogenase (*GAPDH*) expression level as endogenous control. All primers used (Invitrogen) are listed (Table 2).

**Table 2:** Primers used in RT‐qPCR analyses

Genes	Primers	Sequence (5ʹ‐3ʹ)
*NCOA6*	PCR‐F	AAAACGTGCCCAATTTGTTACAC
	PCR‐R	CAATCTGAACGGAGAGAATCCC
*HLA‐G*	PCR‐F	CCATCATGGGTATCGTTGCT
	PCR‐R	GCTCCCTCCTTTTCAATCTG
*ITGA5*	PCR‐F	GTCGGGGGCTTCAACTTAGAC
	PCR‐R	CCTGGCTGGCTGGTATTAGC
*MMP2*	PCR‐F	TACAGGATCATTGGCTACACACC
	PCR‐R	GGTCACATCGCTCCAGACT
*MMP9*	PCR‐F	CAGTCCACCCTTGTGCTCTTC
	PCR‐R	TGCCACCCGAGTGTAACCAT
*TIMP1*	PCR‐F	ACCACCTTATACCAGCGTTATGA
	PCR‐R	GGTGTAGACGAACCGGATGTC
*TIMP2*	PCR‐F	AAGCGGTCAGTGAGAAGGAAG
	PCR‐R	AGGGTTGCCATAAATGTCGTT
*GAPDH*	PCR‐F	ATGGAAATCCCATCACCATCTT
	PCR‐R	CGCCCCACTTGATTTTGG

Abbreviations: F, Forward; R, Reverse.

### 2.11 TF binding site prediction

Three computational algorithms, namely, LASAGNA‐Search 2.0 (https://biogrid-lasagna.engr.uconn.edu/lasagna_search/),^22^ Tfsitescan (http://www.ifti.org/cgi-bin/ifti/Tfsitescan.pl) and Gene2Promoter (http://www.genomatix.de), were utilized to search for binding sites of RELA/p65 in the *MMP9* proximal promoter with the default system parameters.

### 2.12 Dual‐luciferase assay

Cotransfection in HTR‐8/SVneo cells was performed using a wild type (WT) or mutant luciferase vector of the *MMP9* promoter, pRenilla‐TK (as the internal control), and the *NCOA6* siRNAs/ectopic expression vectors of NCOA6 and RELA/p65 or corresponding negative controls. A dual‐luciferase reporter assay kit (Promega) was applied to determine the change in the luciferase activity of cells 48 hours after transfection with a Varioskan^™^ Flash Microplate Reader.

### 2.13 Western blotting

Western blotting was carried out as previously reported.^14^ Primary antibodies against human NCOA6 (PA5‐52071, Invitrogen; 1:1000) and GAPDH (ab181603, Abcam, Cambridge, UK; 1:10 000) were used in an overnight incubation at 4°C with cut membranes including NCOA6 or GAPDH protein, respectively. After incubation with HRP‐conjugated antibody (ab6721, Abcam; 1:10 000), specific NCOA6 and GAPDH bands were detected with a ChemiDoc MP System.

### 2.14 Statistical analysis

All data are represented as the mean ± SD (standard deviation) following three and more independent experiments, and an independent‐samples *t* test in SPSS Statistics 17.0 was used to determine the significance of the differences between treatments and corresponding controls. Statistical significance is indicated mainly by one asterisk (*) for *P* < .05 or two asterisks (**) for *P* < .01.

## 3 RESULTS

### 3.1 NCOA6 is expressed in human placental TC and invasive EVT cells

**Figure 1:**
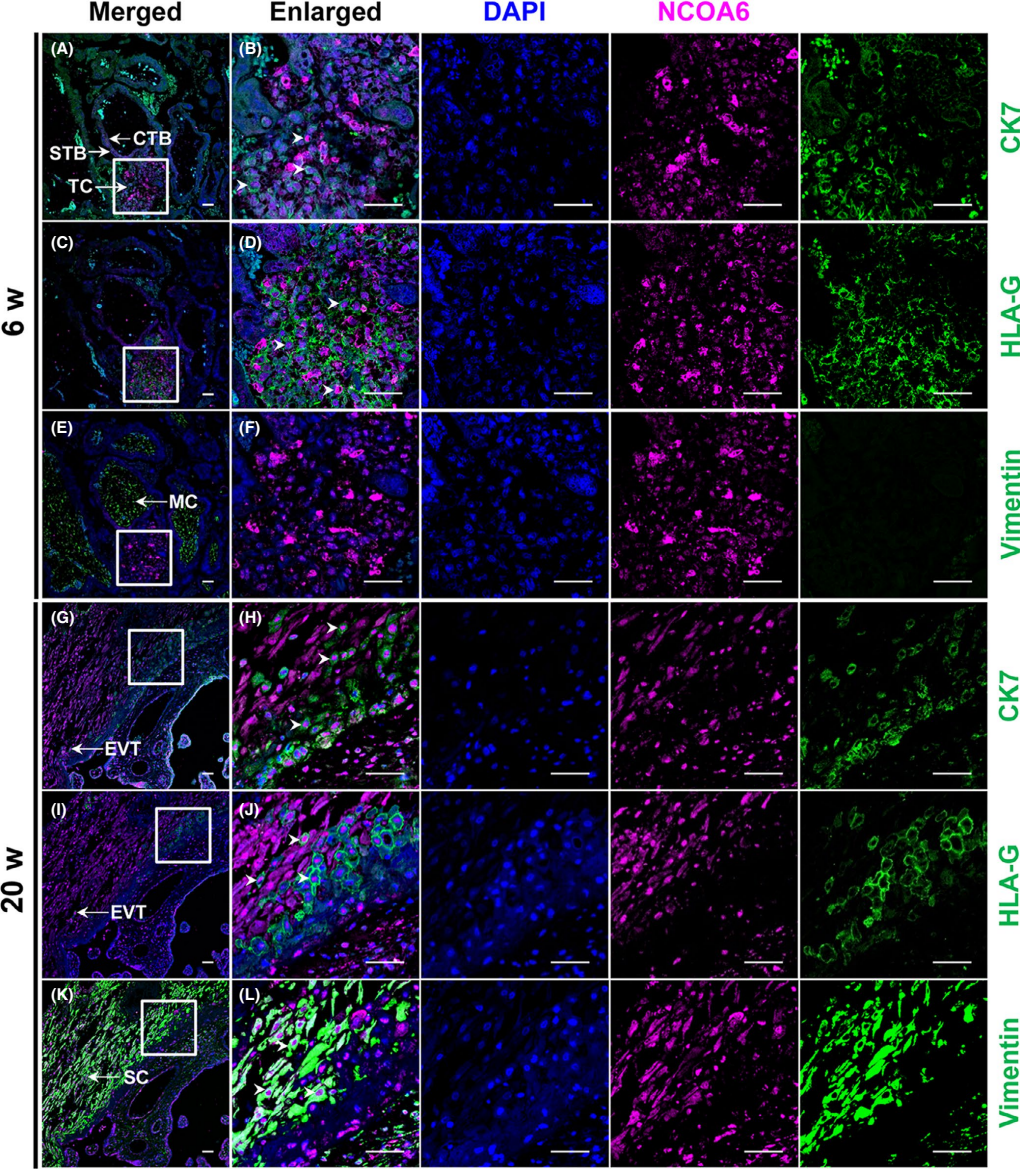
Immunofluorescence localization of NCOA6 in the first‐ and second‐trimester human placental tissues. NCOA6 staining was observed in the CTBs, STBs, TC and EVTs but not in the villous MCs of normal first‐trimester and second‐trimester placentas (A‐J). NCOA6 staining was also found in uterine SCs (K, L). In the representative images of a first‐trimester placental villus from the 6th week of gestation (A‐F), NCOA6 was highly expressed in the TC (C, D). In the representative images of a second‐trimester placenta from the 20th week of gestation (G‐L), strong expression of NCOA6 in the nucleus of EVTs was found (I, J). CK7 was used as the marker for placental trophoblasts; HLA‐G was used as the marker for EVTs; and Vimentin was used as the marker for villous MCs and uterine SCs. Scale bars: 50 μm. CTBs indicates cytotrophoblasts; EVTs, extravillous trophoblasts; HLA‐G, human leucocyte antigen‐G; MCs, mesenchymal cells; SCs, stromal cells; STBs, syncytiotrophoblasts; TC, trophoblast column

**Figure 2:**
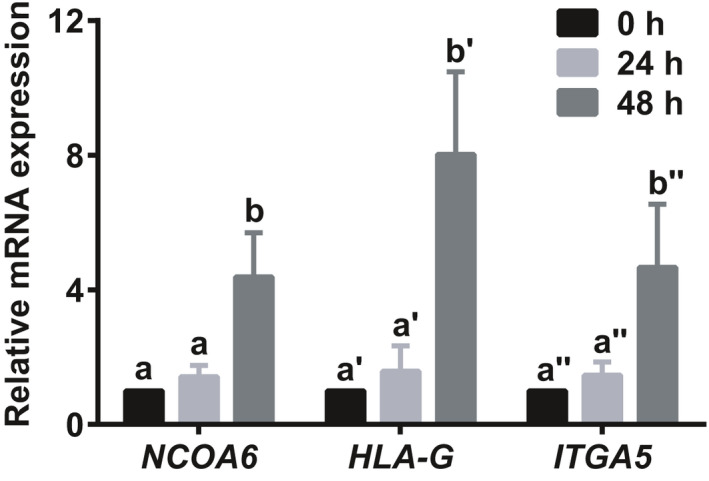
*NCOA6* expression in primary CTBs isolated from the first‐trimester human placental villi was increased during in vitro EVT induction. CTBs were purified from placental villi at 6‐8 weeks of gestation by Percoll gradient and subsequently cultured in 1 mg/mL liquid Matrigel precoated plates to induce differentiation to EVTs. *NCOA6* expression was measured 0, 24 and 48 hours after cell plating. The EVT markers *HLA‐G* and *ITGA5* were used as indicators of EVT induction. The results are presented as the mean ± SD based on four independent experiments. The values with different letters are significantly different (*P* < .05). CTBs indicates cytotrophoblasts; EVT, extravillous trophoblast; HLA‐G, human leucocyte antigen‐G

The expression of NCOA6 in the first‐ and second‐trimester human placental tissues was first examined by immunofluorescence. The colocalization of NCOA6 and human leucocyte antigen‐G (HLA‐G, an EVT cell surface marker)^23^, ^24^ expression indicated that NCOA6 is mainly expressed in TC in the first‐trimester placenta and EVTs in the second‐trimester placenta (Figure 1), which implies the important roles of NCOA6 in trophoblast invasion and migration. Human primary CTBs were further isolated from placental villi with 6‐8 weeks of gestation to examine the change of *NCOA6* transcription during in vitro induction of EVTs. The RNA level of *NCOA6* was also significantly elevated as those of the specific EVT markers *HLA‐G* and integrin subunit alpha 5 (*ITGA5*) during the in vitro induction of primary CTBs into EVTs (Figure 2), confirming the important roles of NCOA6 in trophoblast invasion and migration.

### 3.2 *NCOA6* knockdown attenuates the invasion and migration of HTR‐8/SVneo cells

**Figure 3:**
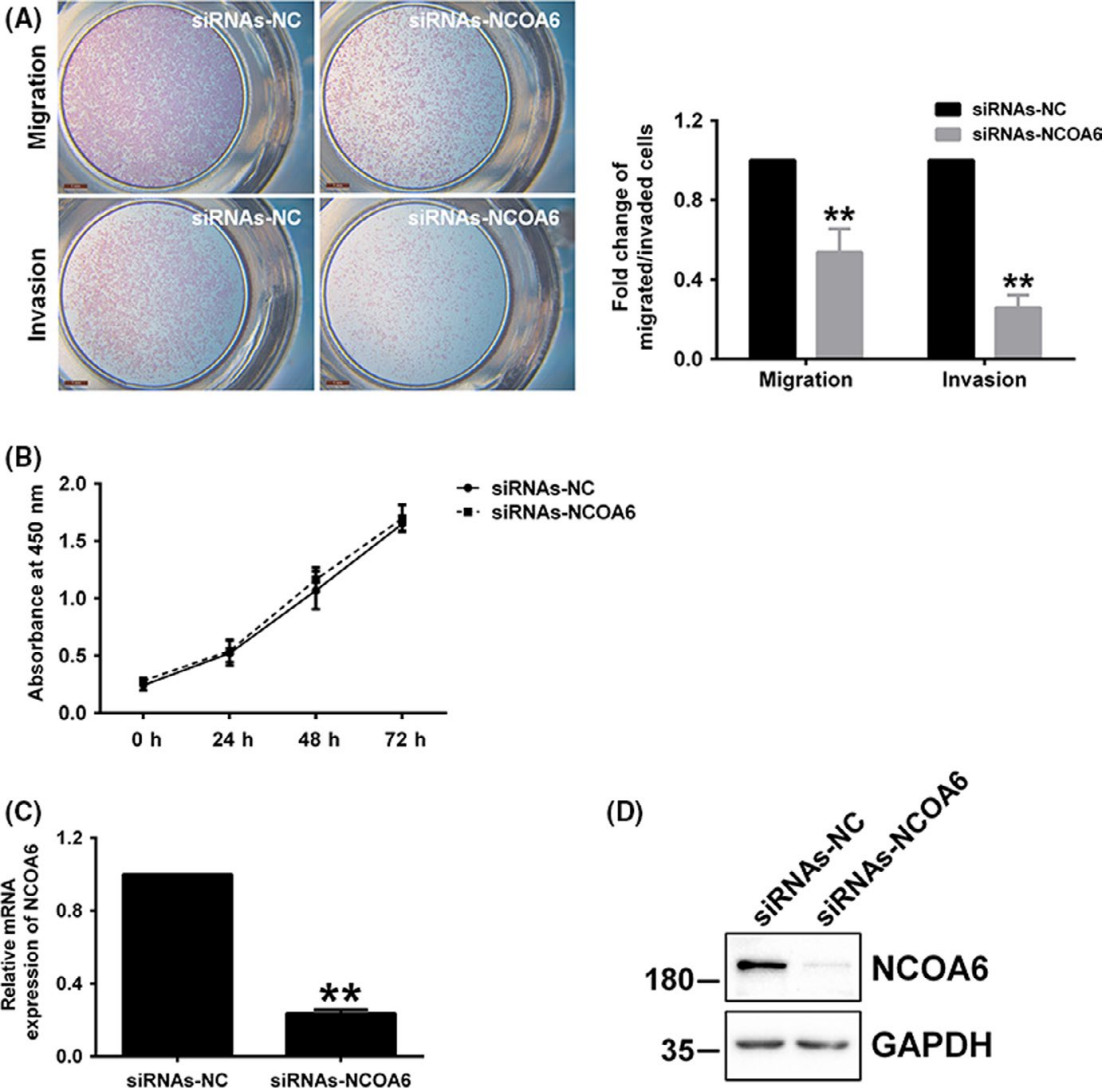
*NCOA6* knockdown significantly impaired the invasion and migration of HTR‐8/SVneo cells. A, Significantly attenuated invasion and migration occurred after the transfection of *NCOA6* siRNAs into HTR‐8/SVneo cells. All invaded/migrated cells outside the insert membrane were stained and photographed (left panel; scale bars: 1 mm). The fold change in cell invasion/migration capacity after treatment was estimated by counting the invaded/migrated cells with staining (right panel). B, In parallel with the transwell assays, CCK‐8 assays were carried out to determine the change in the number of cells 0, 24, 48 and 72 hours after transfection. C, D, The mRNA and protein levels of *NCOA6* were examined after siRNA transfection. A representative image from a Western blot is shown, and the molecular weight markers are indicated on the left in kDa. All results are presented as the mean ± SD based on at least three independent experiments. **P < .01

To further explore whether NCOA6 regulates trophoblast invasion and migration, HTR‐8/SVneo cells (the most commonly applied cell model in trophoblast invasion and migration) were treated with siRNAs targeting *NCOA6* before monitoring cell invasion and migration via transwell assays with/without Matrigel, respectively. HTR‐8/SVneo cell invasion and migration were both significantly attenuated after *NCOA6* siRNA treatment (Figure 3A). To exclude the possibility that cell proliferation during transwell assays might have a secondary effect on the change in the invaded/migrated cell number after *NCOA6* siRNA transfection, cell counting assays were introduced in parallel with transwell assays to record the changes in the number of transfected cells at 0, 24, 48 and 72 hours. *NCOA6* siRNA treatment had no obvious effects on the change in the number of transfected cells (Figure 3B), suggesting that the decreased number of invaded/migrated HTR‐8/SVneo cells after *NCOA6* siRNA treatment was primarily affected by impaired cell invasion and migration capacities. *NCOA6* knockdown by siRNAs was confirmed by RT‐qPCR and Western blotting analyses in parallel (Figure 3C,D).

### 3.3 *NCOA6* knockdown blocks the activation of NF‐κB‐mediated *MMP9* transcription

**Figure 4:**
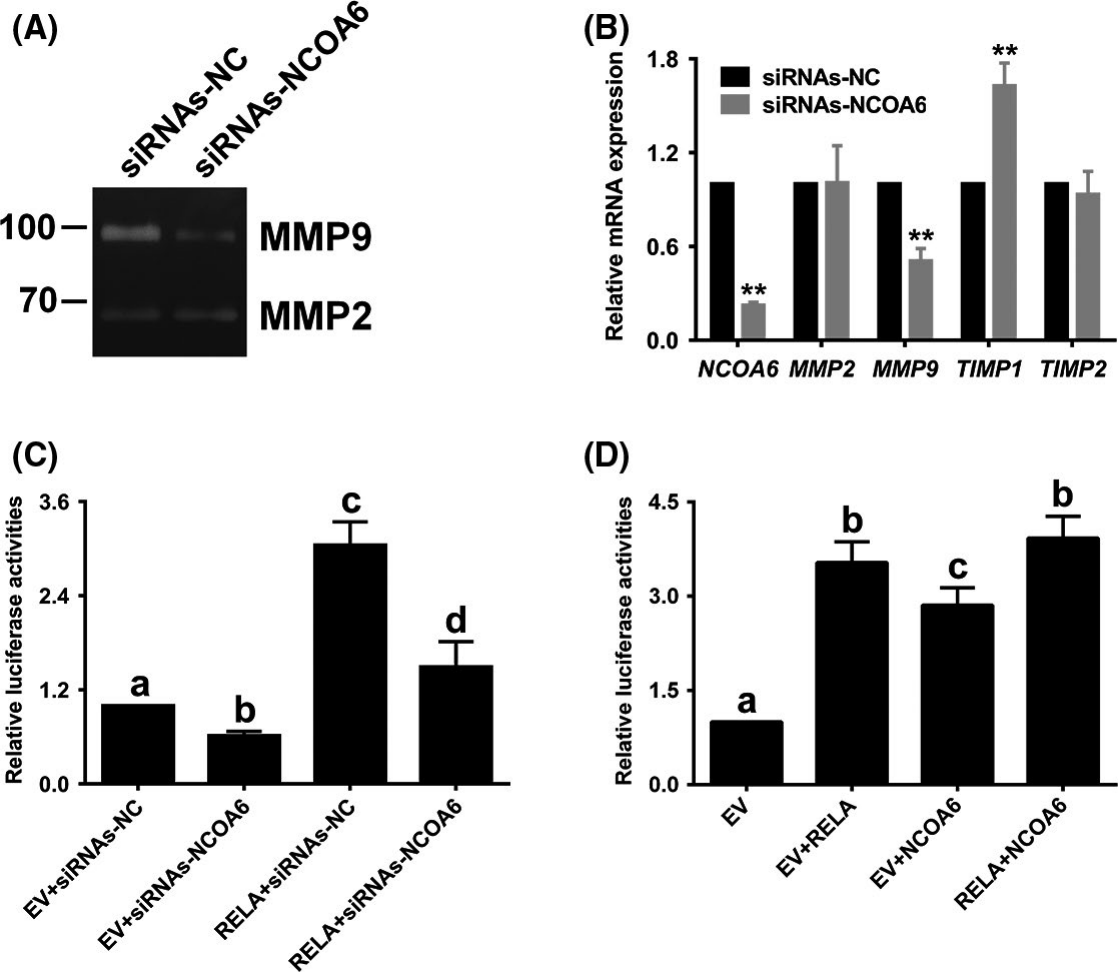
*NCOA6* knockdown blocked the activation of NF‐κB‐mediated *MMP9* transcription. A, The gelatinolytic activity of secreted MMP9, but not of secreted MMP2, was significantly impaired after *NCOA6* was knocked down. B, The transcription of *MMP9* was also significantly attenuated after *NCOA6* was knocked down, while the transcription of *TIMP1* was enhanced. The transcription of *MMP2* and its inhibitor *TIMP2* showed no change. C, Knockdown of *NCOA6* not only significantly decreased the basic reporter activity of the *MMP9* promoter but also blocked the increased reporter activity of the *MMP9* promoter induced by RELA/p65 overexpression in luciferase assays. D, Co‐overexpression of NCOA6 and RELA/p65 did not synergistically enhance the increased reporter activity of the *MMP9* promoter induced by RELA/p65 overexpression alone. All results are presented as the mean ± SD based on at least three independent experiments. **P < .01; the values with different letters are also significantly different (*P* < .05)

Trophoblasts degrade the extracellular matrix (ECM) when they invade and migrate into the maternal decidua and myometrium. Among the various proteinases secreted by trophoblasts, MMP2 and MMP9, belonging to the gelatinase group of the MMP family and being mainly expressed in trophoblasts of early gestation, play essential roles in the invasion and migration of trophoblasts.^25^, ^26^ To investigate whether MMP2 and MMP9 are involved in suppressing the invasion and migration of *NCOA6*‐knockdown HTR‐8/SVneo cells, gelatin zymography assays were performed to examine the changes in the gelatinolytic activities of MMP2 and MMP9. As a result, MMP9 activity was significantly decreased after *NCOA6* was knocked down; however, no change in MMP2 activity was found (Figure 4A). Tissue inhibitors of metalloproteinases (TIMPs) inhibit most MMP activities in tissues with a certain degree of specificity.^26^ Interestingly, in addition to the reduced level of *MMP9* transcription, the transcription of *TIMP1*, which has been reported to preferentially bind MMP9,^27^, ^28^ was increased, while the transcription of *TIMP2*, which preferentially binds MMP2,^28^, ^29^ was not changed, in accordance with the *MMP2* results (Figure 4B). These data suggested the important role of MMP9 but not MMP2 in regulating the invasion and migration mediated by NCOA6 in HTR‐8/SVneo cells.

Since NCOA6 is a transcription coactivator of NF‐κB,^10^ which has been reported to regulate *MMP9* transcription via the binding of its subunit RELA/p65 to *MMP9* promoter,^20^, ^30^, ^31^ we further explored whether NCOA6 coactivates NF‐κB‐mediated *MMP9* transcription using luciferase reporter assays. Knockdown of *NCOA6* not only significantly decreased the basic reporter activity of the *MMP9* promoter but also blocked increased reporter activity of the *MMP9* promoter following RELA/p65 overexpression (Figure 4C). However, compared with RELA/p65 overexpression alone, the co‐overexpression of NCOA6 and RELA/p65 did not synergistically enhance the increased reporter activity of the *MMP9* promoter (Figure 4D). These results further demonstrate that the coactivating role of NCOA6 is essential for NF‐κB‐mediated *MMP9* transcription.

### 3.4 NF‐κB‐mediated transcription occurs via two binding sites in the proximal promoter of *MMP9*

**Figure 5:**
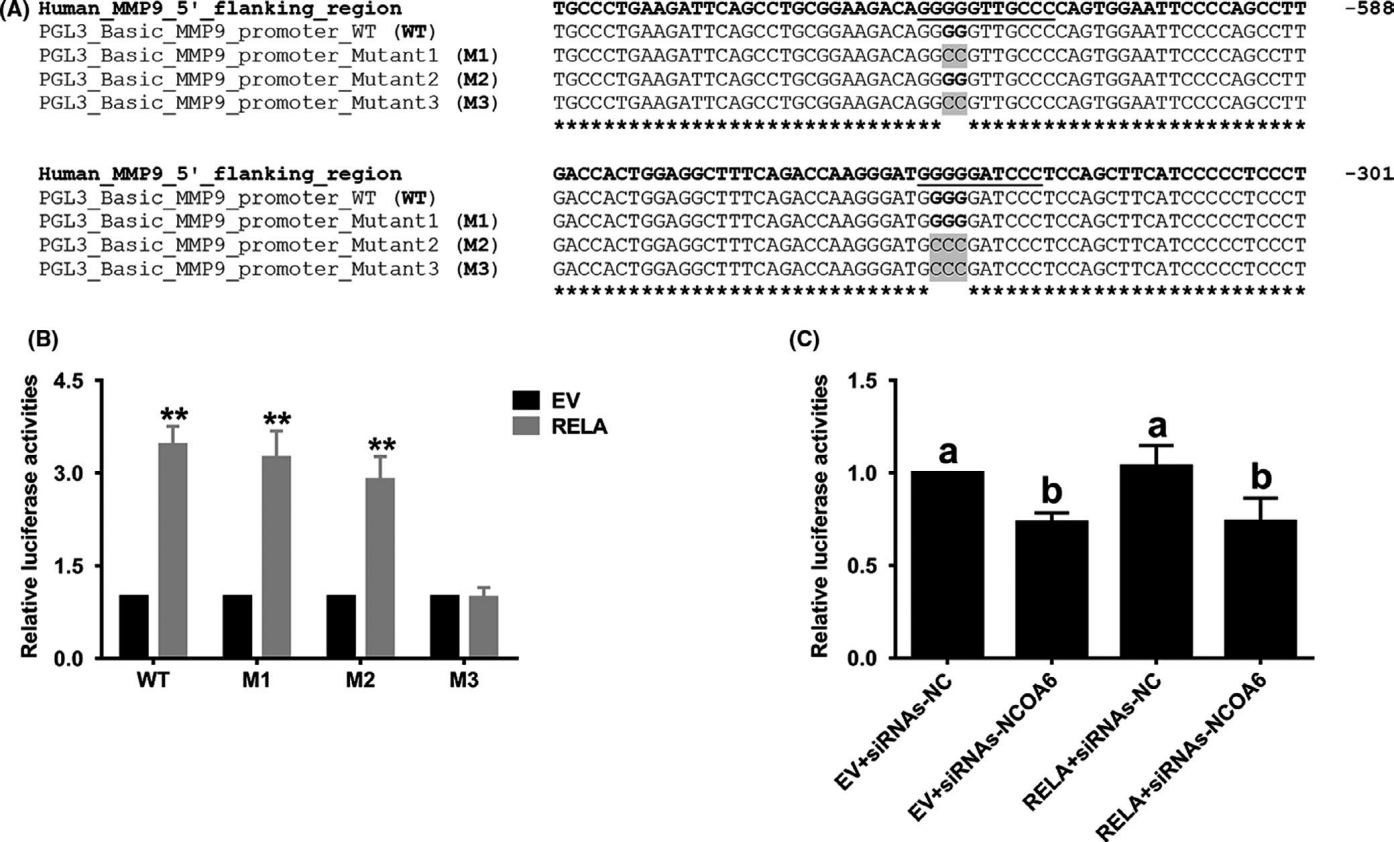
Two binding sites of NF‐κB in the *MMP9* proximal promoter mediate its transcriptional regulation. A, Two putative binding sites of NF‐κB are shown in the proximal promoter of *MMP9*. B, Only the M3 mutant promoter, but neither M1 nor M2, could completely interrupt NF‐κB‐mediated *MMP9* transcription. C, Reporter activity of M3 was still significantly decreased after *NCOA6* knockdown. All presented results are expressed as the mean ± SD based on at least three independent experiments. **P < .01; the values with different letters are also significantly different (*P* < .01)

Three computational algorithms, namely, LASAGNA‐Search 2.0, Tfsitescan and Gene2Promoter, were utilized to search for putative binding sites of NF‐κB in the *MMP9* proximal promoter. Two binding sites were found: one site was located at −619 to −609 bp as reported previously,^20^ and the other site was located at −331 to −322 bp (Figure 5A). These two sites were individually or both mutated, and the mutations were referred to as the M1 mutation (for site −619 to −609), the M2 mutation (for site −331 to −322) and the M3 mutation (for both sites) (Figure 5A). Only the M3 mutation in the promoter was found to completely interrupt NF‐κB‐mediated *MMP9* transcription (Figure 5B), suggesting that both binding sites in the proximal promoter function in NF‐κB‐mediated *MMP9* transcription. However, the knockdown of *NCOA6* still significantly reduced the reporter activity of M3 (Figure 5C) with less fold change than that of WT affected by *NCOA6* knockdown (Figure 4C), which suggested that in addition to NF‐κB, NCOA6 might coactivate other transcription factor(s) that bind to the proximal promoter of *MMP9*, for example, AP‐1,^10^, ^20^ during the invasion and migration of HTR‐8/SVneo cells.

### 3.5 Transcript levels of *NCOA6* were decreased in early‐onset sPE placentas

**Figure 6:**
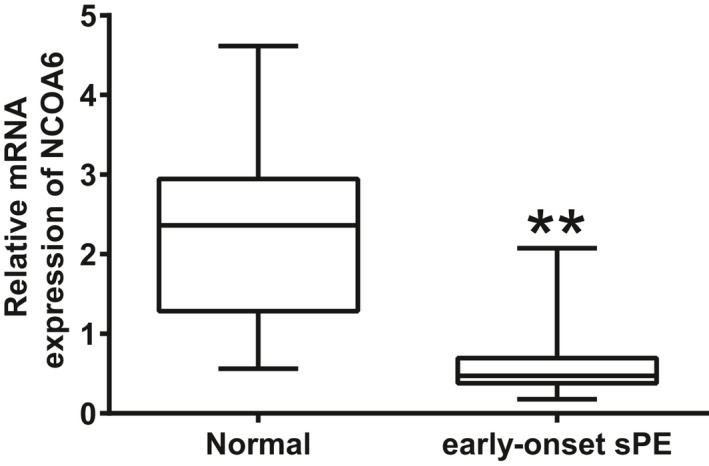
Transcription of *NCOA6* was significantly impaired in early‐onset sPE placentas. Transcription of *NCOA6* in early‐onset sPE placentas (n = 13) was compared with that in paired normal placentas (n = 15) by RT‐qPCR. **P < .01

Since inadequate EVT invasion and migration primarily contribute to early‐onset PE, the transcript levels of *NCOA6* in the placentas of pregnancies with early‐onset sPE were examined to evaluate whether placental transcription of *NCOA6* is also altered in this diseased state. Of interest, transcript levels for *NCOA6* in the early‐onset sPE placentas were significantly impaired compared with those in the paired placentas from normal pregnancies (Figure 6).

## 4 DISCUSSION

After embryo implantation, the development of the foetus is supported by the placenta, where nutrients and oxygen are provided while waste and carbon dioxide are removed.^32^ During pregnancy, the spiral arteries that ultimately supply the placenta are reconstructed by part of trophoblast cells that have invaded and migrated into the maternal decidua/myometrium and further the maternal vessels. Shallow trophoblast invasion and migration are associated with many pregnancy‐specific complications, including PE.^33^

NCOA6 is a transcription coactivator that executes a variety of functions with specific transcription factors including PPARα, PPARγ, AP‐1, CRE and NF‐κB.^8^, ^9^, ^10^ The deletion of *Ncoa6* in mice resulted in developmental defects in the placenta and lethality between 8.5 and 12.5 dpc^9^, ^11^, ^12^; however, the mechanisms involved remain largely unknown. In the present work, NCOA6 was found to be expressed in the TC and invasive EVTs of human placenta. In addition, the RNA level of *NCOA6* was also increased as those of the specific EVT markers *HLA‐G* and *ITGA5* during the in vitro induction of isolated primary CTBs into EVTs. These results suggested that NCOA6 is required for trophoblast invasion and migration. Due to the restricted culture days of primary trophoblasts, HTR‐8/SVneo cell line was then applied to investigate the functions of NCOA6 in trophoblast invasion and migration. HTR‐8/SVneo cell line, the most commonly used cell model in trophoblast invasion and migration, was established by stably transfecting the gene encoding simian virus 40 (SV40) large T antigen into cultured first‐trimester human trophoblasts. Apart from their extended lifespans, HTR‐8/SVneo cells possess many similar phenotypic/functional properties with the primary trophoblast cells, for example in vitro morphology, in vitro invasive abilities, and secrection of type IV collagenase.^18^ As expected, the knockdown of *NCOA6* significantly attenuated HTR‐8/SVneo cell invasion and migration.

Before trophoblasts invade and migrate into the maternal decidua and myometrium, the ECM, which is important for creating the cellular environments of uterine tissue, must be degraded and remodelled. MMP family is involved in tissue remodelling and angiogenesis, with 23 members identified to date.^34^ Nearly all human MMPs, specifically secreted by uterine natural killer cells, decidual cells and placental trophoblasts, are present at the human fetomaternal interface^35^; among them MMP9, but not MMP2, has been reported to exhibit reduced expression in placentas complicated by PE.^36^, ^37^ In addition, deficiencies in trophoblast invasion emerged soon after implantation in *Mmp9*‐null mouse embryos, and clinical features of PE were observed in pregnant *Mmp9*‐null mice with *Mmp9*‐null embryos.^38^ These results demonstrated the crucial roles of MMP9 in trophoblast invasion during human placentation. We found in this study that the gelatinase activity of secreted MMP9, but not that of secreted MMP2, was significantly impaired after *NCOA6* siRNA treatment.

NF‐κB, one of the transcription factors that can be coactivated by NCOA6, has been reported to regulate *MMP9* transcription via the binding of its subunit RELA/p65 to the promoter.^20^, ^30^, ^31^ We found in this study that *NCOA6* knockdown not only significantly decreased the basic reporter activity of the *MMP9* promoter but also prevented the increased reporter activity of the *MMP9* promoter induced by RELA/p65 overexpression, suggesting an essential role of NCOA6 in the coactivation of NF‐κB‐mediated *MMP9* transcription. However, co‐overexpression of NCOA6 and RELA/p65 did not promote increased reporter activity of the *MMP9* promoter induced by RELA/p65 overexpression alone.

Two binding sites of NF‐κB on the *MMP9* proximal promoter, including one (at −619 to −609 bp) reported previously,^20^ were bioinformatically predicted, and both sites were validated by site mutagenesis. However, the activity of the reporter with both binding sites mutated remained significantly decreased after *NCOA6* knockdown, suggested the presence of other NCOA6‐coactivated transcription factors, for example AP‐1,^10^, ^20^ that bind to the proximal promoter of *MMP9* in HTR‐8/SVneo cells.

Since NCOA6 has essential roles in trophoblast invasion and migration and inadequate trophoblast invasion/migration and subsequent incomplete reconstruction of maternal spiral arteries are the main contributors to the early‐onset PE,^2^, ^3^, ^4^, ^39^ the transcript levels of *NCOA6* were compared in placentas from pregnancies with early‐onset sPE and normal pregnancies. Notably, early‐onset sPE placentas showed significantly fewer *NCOA6* transcripts than normal placentas. The transcriptomics of CTBs have been found to be dysregulated in sPE due to its diseased in vivo environment.^40^ As addressed above, NCOA6 is a transcription coactivator that executes a variety of functions with many specific transcription factors. These results imply transcription coactivators including NCOA6 might play critical roles in the pathogenesis of sPE. However, one limitation exists in this section: placental tissues from normal pregnancies and pregnancies complicated by early‐onset sPE were not completely “term‐matched” (Table 1). Significant difference in genstational age might partly contribute to the decreased *NCOA6* transcript levels observed in early‐onset sPE placentas.

In conclusion, our results demonstrated that NCOA6, a transcription coactivator, promotes the invasion and migration of trophoblasts, at least partially, by activating NF‐κB‐mediated *MMP9* transcription. The “in vivo” functions of NCOA6 in placentation and the roles of downregulated NCOA6 in sPE development need to be investigated in the future; for example, placenta‐specific *Ncoa6* knockout mice should be constructed and analysed to study the “in vivo” function of NCOA6 in placental development. Furthermore, additional researches are also needed to verify other transcription factors that are coactivated by NCOA6 during placentation and sPE development.

## CONFLICT OF INTEREST

The authors have no conflict of interest to declare.

## AUTHOR CONTRIBUTIONS

The contributions each author made to the study are specified as follows: YPS and LW designed the study; LW, KQZ, WW, LNC and JS performed the experiments and collected the data; YPS, LW, KQZ, WW, LLH and XXJ analysed the data; LW and KQZ prepared the manuscript; YPS and LW revised the manuscript.

## Data Availability

The data that support the prediction of RELA/p65 binding sites in the *MMP9* proximal promoter in this study are available from LASAGNA‐Search 2.0 (https://biogrid-lasagna.engr.uconn.edu/lasagna_search/),^22^ Tfsitescan (http://www.ifti.org/cgi-bin/ifti/Tfsitescan.pl) and Gene2Promoter (http://www.genomatix.de), with the default parameters.
